# Bi-directional exosome-driven intercommunication between the hepatic niche and cancer cells

**DOI:** 10.1186/s12943-017-0740-6

**Published:** 2017-11-14

**Authors:** Nikolina Dioufa, Amanda M. Clark, Bo Ma, Colin H. Beckwitt, Alan Wells

**Affiliations:** 1 Department of Pathology, University of Pittsburgh, Pittsburgh, PA USA; 2 McGowan Institute for Regenerative Medicine, University of Pittsburgh, Pittsburgh, PA USA; 3 University of Pittsburgh Cancer Institute, Pittsburgh, PA USA; 4 Pittsburgh VA Medical Center, VA Pittsburgh Healthcare System, S713 Scaife Hall, 3550 Terrace St, Pittsburgh, PA 15261 USA

**Keywords:** Exosomes, Microphysiological system, E-cadherin, Mesenchymal-to-epithelial-reverting-transition

## Abstract

**Background:**

Our understanding of the multiple roles exosomes play during tumor progression is still very poor and the contribution of the normal tissue derived exosomes in distant seeding and tumor outgrowth has also not been widely appreciated.

**Methods:**

Using our all-human liver microphysiological system (MPS) platform as a model to closely recapitulate the early metastatic events, we isolated exosomes from both tumor cells and liver microenvironment.

**Results:**

We observed that while priming of the hepatic niche (HepN) with MDA-231 breast cancer derived exosomes facilitated seeding of the cancer cells in the liver, subsequent tumor outgrowth was diminished; this was consistent with increased entry into dormancy. We found that hepatic niche (HepN) derived exosomes contribute significantly to the exosome pool and are distinguished from cancer derived exosomes based on their size, protein and miRNA content. By Ingenuity Pathway Analysis (IPA) of the miRNA content of the HepN, MDA-231/HepN and MDA-231 cells we showed that the HepN derived exosomes affect the breast cancer cells by suppressing pathways involved in cancer cell proliferation and invasion. More importantly exposure of MDA-231 and MDA-468 cells to purified normal HepN derived exosomes, induced changes in the cells consistent with a Mesenchymal to Epithelial reverting Transition (MErT). miRNA arrays performed on MDA-231 treated with Hum Hep/NPC derived exosomes showed significant changes in the levels of a select number of miRNAs involved in epithelial cell differentiation and miRNAs, such as miR186, miR23a and miR205, from our top and bottom bins have previously been reported to regulate E-cadherin transcription and MErT induction in various cancer types. Consistently HepN derived exosome treatment of breast and prostate cancer lines lead to a transient induction of E-cadherin and ZO-1 at the protein level and a more epithelial-like morphology of the cells.

**Conclusions:**

Collectively our data revealed a novel mechanism of regulation of the metastatic cascade, showing a well-orchestrated, timely controlled crosstalk between the cancer cells and the HepN and implicating for the first time the normal tissue/HepN derived exosomes in enabling seeding and entry into dormancy of the cancer cells at the metastatic site.

**Electronic supplementary material:**

The online version of this article (10.1186/s12943-017-0740-6) contains supplementary material, which is available to authorized users.

## Background

During her lifetime, one in eight women will be diagnosed with breast cancer and despite the multimodal therapeutic schemes available, metastatic disease remains the main cause of mortality. Over half of those that succumb will have overt liver metastases [1]. Metastasis is a multistep process that requires detachment of the cancer cells from the local tumor, extravasation and survival in the blood circulation, followed by an ectopic survival and proliferation at a distant site. The initial step for cells to leave the primary tumor is the loss of epithelial cell–cell adhesions and subsequent acquisition of a more mesenchymal-like invasive phenotype, referred to as Epithelial–Mesenchymal Transition (EMT) [2], main hallmark of which is loss of E-cadherin, a calcium (Ca^2+^)-dependent transmembrane glycoprotein that mediates cell-cell cohesion. However, we and others have shown that while necessary for invasion, E-cadherin downregulation is reversed during the metastatic seeding [3–5] and that entry into dormancy relies on this cancer cell phenotypic plasticity through which highly dedifferentiated breast cancers appear more epithelial in the metastatic milieu [2, 6–8]. Our laboratory has focused on the development and use of an all-human liver microphysiological system (MPS) that uses fresh human hepatic tissue to recapitulate the hepatic niche (HepN) so as to study the steps of the metastatic seeding, tumor outgrowth and entry into dormancy of cancer cells as a model that closely mimics the process in vivo and allows the monitoring and immediate interactions between the cancer cells and the hepatic microenvironment [9–11].

How cancer cells communicate with their local and distant environment is undergoing a re-evaluation with the discovery that all cells release extracellular vesicles (EVs) to their surrounding milieu. EVs vary in size and function from apoptotic bodies (1000–5000 nm) to microvesicles (MVs 200–1000 nm) and exosomes (30-150 nm). Exosomes were first described in the early 1980s during reticulocyte maturation and were believed to be a form of “cellular waste disposal”. It took several decades for the scientific community to realize that the exosomes are critical to tissue homeostasis and master regulators of cell-to-cell communication [12, 13]. Contrary to the other EVs formation process, the biogenesis of exosomes is intricate and requires multiple levels of regulation, including the formation of late endosomes or multivesicular bodies (MVBs). MVB formation is primarily regulated by the endosomal sorting complexes required for transport (ESCRT) and during this stage, cytoplasmic RNA and proteins get incorporated into exosomes. Subsequently these MVBs fuse with the cell membrane and release exosomes to the extracellular space [14]. All exosomes contain common proteins that serve as exosome markers during their isolation, including the tetraspanins CD9, CD81 and CD63, ESCRT associated proteins, like Alix and TSG101 and cytoplasmic proteins, such as Hsp70. However, the cell of origin and its physiologic state at any given time will also be reflected in the exosomal components, in addition to those structural ones noted above.

Through their cargo DNA, mRNA, miRNA, enzymes and soluble factors, exosomes can mediate various autocrine and paracrine signals and orchestrate intercellular communication. More specifically tumor derived exosomes or oncosomes [13] have largely been described as promoters of tumor progression and drivers of metastasis by generating a premetastatic milieu through fibroblast activation, immune suppression, extracellular matrix remodeling, enhanced vasculogenesis and/or drug resistance [13, 15–17]. Even though our understanding of the oncosomal contribution during tumor progression has advanced during the last decade, all our information is extracted from 2D cultures that do not closely mimic the metastatic cascade as it unfolds in the human body. Moreover, even though normal exosomes have been shown to be important for tissue homeostasis and wound healing, very little is known about the effects of normal tissue exosomes on tumor cells, particularly in reference to distant seeding and tumor outgrowth during cancer progression.

Herein, we studied the effect of exosomes in the metastatic seeding and proliferation of breast cancer cells to the liver. We showed that priming of the HepN with breast cancer derived exosomes results in significant enhancement of the cancer cell seeding in the liver, followed however by a suppressed tumor cell outgrowth in proliferation experiments. The same data was recapitulated in our Liver MPS that closely mimics the metastatic process. We hypothesized that the suppressed proliferation was due to the effect of the HepN derived exosomes on the colonizing breast cancer cells. We validated that the HepN significantly contributes to the exosome pool and that these exosomes, significantly different in size, protein and miRNA content, through miRNA regulation, activate pathways that suppress cancer cell proliferation and invasion. More importantly we show that treatment of MDA-231 and MDA-468 with purified normal HepN derived exosomes increases E-cadherin and ZO-1 protein expression levels in both breast cancer lines. Our data identifies a novel mechanism of regulation of the metastatic cascade, introducing the normal tissue/HepN derived exosomes as significant modulators of MErT during seeding and suppression of tumor growth once the breast cancer cells have reached the liver.

## Methods

### Cell lines and culture

Breast cancer cell lines, MDA-231 and MDA-468, were cultured in RPMI medium. MDA-231 obtained from ATCC were stably transfected with Red Fluorescent Protein (RFP) expressing vector as previously described [8]. Prostate cancer line DU145 was cultured in DMEM medium. The melanoma line WM35 was maintained in MCDB153: L15 4:1 medium mixture with addition of 5% Fetal Bovine Serum (FBS), 5 μg/ml insulin and 2 mM CaCl_2_. The WM852 melanoma line was cultured in DMEM:L15 3:1 mixture with addition of 10% FBS [18]. TPF16238, melanoma cells derived from patient sample, were kindly provided by Dr. Kirkwood and maintained in RPMI with 1 mM L-glutamine, 0,1 mM NEEA and 10 nM HEPES. All cells were incubated at 37 °C in 5% CO_2_, and supplemented with 1% *v*/v penicillin/streptomycin, and 10% v/v FBS, unless specified otherwise (Gibco, Life Technologies, Grand Island, NY, USA). The Trypan Blue exclusion method was used to assess cell viability. As FBS contains exosomes of bovine origin, all cell cultures were depleted of serum for 24 h prior to exosomes isolation as previously described by *Théry* et al. [19].

### Liver cells

The primary human hepatocytes (Hep) and non-parenchymal cells (NPCs) were obtained from therapeutic partial hepatectomies for metastatic colorectal carcinoma or, more usually, benign diseases such as focal nodular hyperplasia and hemangiomas. The cells are available from the NIDDK-funded Liver Tissue and Cell Distribution System (LTCDS) with the procurement core directed by Dr. David Geller at the University of Pittsburgh and funded by the NIH (Contract #HHSN276201200017C). The livers are perfused and separate isolations of Hep and NPCs were provided to us, as previously described [20]. We further process the NPC fraction (to eliminate contaminating debris, hepatocytes, and red blood cells) as previously reported [21].

### Exosome isolation

Exosomes were purified from cell culture supernatants by ultracentrifugation as previously described [15]. Briefly, FBS free culture medium was collected and centrifuged at 300×g for 10 min to remove whole cells. The supernatant was then centrifuged at 3,000×g for 20 min to remove dead cells and debris. This supernatant was centrifuged at 10,000×g for 30 min to further remove cell debris. This supernatant was then spun at 100,000×g for 70 min and the pellet was washed with excess PBS to remove contaminating proteins followed by a 70 min centrifugation at 100.000×g to obtain the exosome pellet.

Isolation of exosomes from the liver MPS was performed using the Total Exosome Isolation Reagent from cell culture media (Life Technologies); this method allowed for more efficient handling of smaller volumes from the MPS. After a 20 min centrifugation at 3,000×g the supernatant, containing the exosomes, was removed and combined with 1× volume of the Total Exosome Isolation Reagent and incubated overnight at 4 °C. The exosomes were harvested after a 60 min centrifugation step at 10,000×g. The exosome pellet was subsequently washed in Phosphate Buffered Saline (PBS) followed by a 70 min spin at 100.000×g. A bicinchoninic acid (BCA) protein assay kit (Pierce, Thermo Fisher, OH, USA) was used to determine the concentration of exosome proteins and performed as per the manufacturer’s instructions.

### Transmission electron microscopy

5 μl of freshly isolated exosomes in PBS suspension were applied to copper mesh Formvar coated carbon stabilized grids. They were allowed to adsorb to the grid for 2-3 min and then were wicked off with filter paper. For negative staining of the exosomes, 1% Aqueous Uranyl Acetate (5-10 μl) was applied to the grid for 30 s, then wicked off with Whatman filter paper. Grids were allowed to thoroughly dry before viewing.

### Exosome staining-functional assay

Freshly isolated exosomes were stained with the red lipophilic dye DiI (Thermo Fisher, OH, USA) which is incorporated in the outer exosome membrane. Exosomes were stained in the dark at room temperature in 1 μM DiI, then washed in PBS and centrifuged for 70 min at 100,000×g to remove the unincorporated dye. Pelleted exosomes were resuspended in PBS and added to the cells in culture.

### Exosome RNA isolation, cDNA and miRNA analysis

After the last PBS wash the exosome pellet was resuspended in RLT buffer and total RNA isolation was performed using the RNeasy kit (Qiagen, Hilden, Germany). RNA concentration was measured using the NanoDrop2000 Spectrophotometer (yield range: 60-160 ng/μl). For miRNA analysis, the Cancer Focus microRNA PCR Panel, miRCURY LNA was used (Exiqon, Denmark) by following the manufacturer’s protocol, and using the ExiLENT SYBR® Green master mix. Prior to amplification RNA was converted to cDNA with the Universal cDNA Synthesis Kit II (Exiqon, Denmark).

### Plasmids and reagents

The pCT-CD63-GFP vector was obtained from System Bioscience (SBI, Palo Alto, CA, USA) and MDA-231 RFP+ cells were transfected using Lipofectamine2000 (Life Technologies, Grand Island, NY, USA) using 2,5 μg of plasmid DNA.

### Ex vivo liver MPS

The ex vivo Liver MPS is assembled as recommended by the manufacturer (CN Bio Innovations Ltd., Oxford, UK). Scaffolds for tissue growth are freshly coated with 1% rat tail collagen type I (BD Biosciences, Life Technologies, Grand Island, NY, USA) in PBS for 1 h at 37 °C and washed with PBS before placement in the system. Prior to seeding the system is primed with 1% Bovine Serum Albumin (BSA) at 37 °C, which is then replaced with William’s Medium E (Gibco, Life Technologies) supplemented with the Hepatocyte Thawing and Plating Supplement Pack (Life Technologies). To recapitulate physiologic conditions hepatocytes and NPCs are plated at a 1:1 ratio (6 × 10^5^ cells per well) in the system. Hepatocytes are cultured for approximately 16 h in the plating William’s E medium, described above, and are subsequently changed to William’s E medium supplemented with the Hepatocyte Maintenance Supplement Pack (Life Technologies), prepared following the manufacturer’s instructions. The medium is exchanged completely every 48 h. On day 2 and day 3 the system is primed using cancer derived exosomes (20 μg/well/day) and on day 3 cancer cells are introduced into the formed liver tissue. For seeding into the Liver MPS, 500 MDA-231 RFP+ cells were trypsinized and neutralized in complete growth medium, centrifuged and resuspended in hepatocyte maintenance medium.

### 2D co-culture experiments

6 × 10^5^ primary human hepatocytes were plated using William’s Medium E supplemented with the Hepatocyte Thawing and Plating Supplement Pack in a well of a 6-well plate coated with 1% rat tail collagen in PBS and were allowed to attach overnight. The following day the medium was changed to William’s Medium E supplemented with the Hepatocyte Maintenance Supplement Pack. On day 2 and day 3 the cultures received cancer derived exosomes (20 μg/well/day) for priming and on day 3, 500 RFP+ cancer cells were seeded onto the hepatocyte monolayers. For the transwell assay, after priming of the HepN on day 2 and day 3, 2 × 10^4^ cells were plated on the transwell coculture inserts (Millipore).

### Immunofluorescence microscopy/ cancer cell detection and enumeration

MDA-231 and MDA-468 breast cancer cells were seeded on coverslips precoated with 1% collagen and then fixed with 2% paraformaldehyde in PBS. Following cell permeabilization with 0.1% Triton X-100, non-specific epitopes were blocked with 2% BSA and the cells were incubated with primary antibody diluted in 0.5% BSA at 4 °C overnight. After 2 washes in 0.5% BSA, cells were stained with Alexa Fluor® 488 conjugated secondary antibody at room temperature for 1 h. DAPI was applied to stain the nucleus.

For the 2D co-culture experiments, cells were fixed on day 4 and day 7 with 2% paraformaldehyde in PBS for 1 h at 4 °C and then were visualized using an Olympus BX51 with a 4× (PlanApo NA.0.08) objective (Olympus America Inc.). Digital images were obtained on an Olympus CCD camera using the SPOT 5.2 image acquisition software. The level of RFP-expressing breast cancer cells was determined using the ImageJ software. Images from the entire scaffolds were inclusively thresholded and the RFP+ portion measured as a percentage of the total area.

### Western blot

Exosomes and cells were lysed using radioimmune precipitation assay (RIPA) buffer with phosphatase inhibitor mixture II, III (Sigma) and protease inhibitor mixture (BD Biosciences). Protein extracts, separated by SDS-PAGE and transferred onto PVDF membranes, were probed with antibodies against E-cadherin (BD Biosciences), ZO-1 (BD Biosciences), CD81 and CD63 (System Bioscience) or actin (Sigma). Proteins of interest were detected with HRP-conjugated secondary anti-mouse IgG antibody (Sigma, 1:10.000) and visualized with the Pierce ECL Western blotting substrate (Thermo Scientific, Rockford, IL), according to the provided protocol. The Exo-Check™ exosome antibody arrays were purchased from System Bioscience and performed as instructed by the manufacturer.

### Cell cycle analysis

Samples were collected and fixed in 70% ethanol overnight at 4 °C. For cell cycle analysis, fixed cells were washed in PBS and treated with RNase for 20 min at 37 °C. 5 μg/mL of Propidium Iodide (PI) were added to each sample and analyzed by flow cytometry.

### IPA

The pathway analysis for the miRNA data was done with the use of QIAGEN’s Ingenuity Pathway Analysis (QIAGEN Redwood City, https://www.qiagenbioinformatics.com/products/ingenuity-pathway-analysis/, IPA Winter Release 2014).

### Statistical analysis

Statistical analysis for all experiments was performed with using Prism7 software (GraphPad, La Jolla, CA). An independent Student’s t-test was used to determine statistical differences between experimental and control values. *P* values <0.05 were considered statistically significant.

## Results

### Isolation and identification of functional breast cancer exosomes (breast oncosomes)

**Fig. 1:**
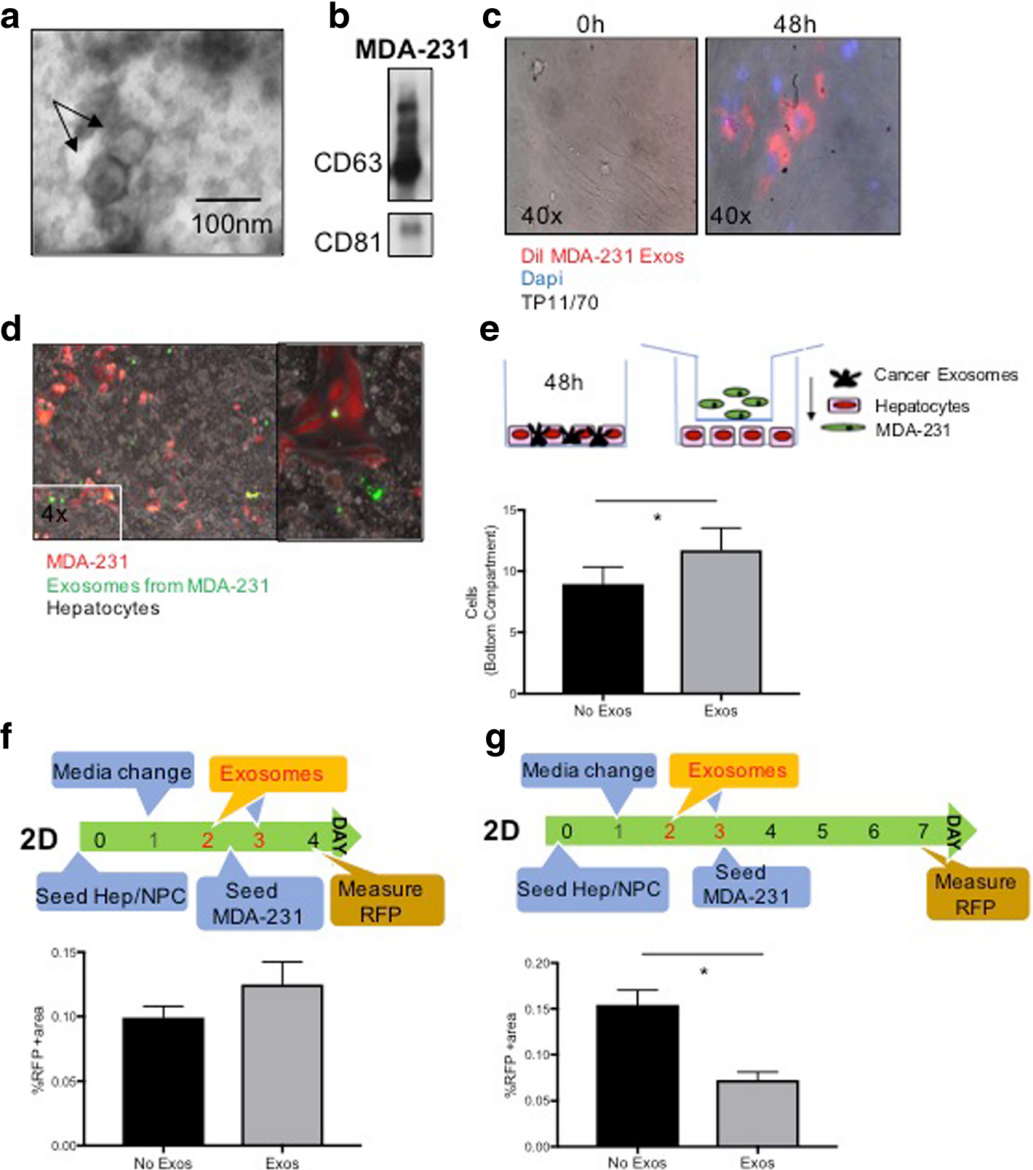
Breast cancer cell produce exosomes that interact with and alter human hepatocytes. **a** Transmission electron microscopy of concentrated exosomes in the media of MDA-231 cells. **b** Immunoblot analyses of the exosome-specific markers CD81, CD63 in MDA-231 exosome protein extracts. **c** MDA-231-derived exosomes were stained with DiI and overlaid on top of normal human fibroblasts TP11/70. The cells were then washed and fluorescence in the cells was assessed at 0 and 48 h. After fixing the cells at 48 h, DAPI was used for nuclear staining. **d** MDA-231 cells expressing RFP were transfected with CD63-GFP. These cells were cocultured with human hepatocytes, with the GFP showing uptake of exosomes distant from the RFP+ MDA-231 cells. Right panel shows higher magnification. **e** MDA-231 cells migrate towards exosome-primed hepatocytes. Human hepatocytes were seeded on the bottom of a transwell plate and primed with MDA-231-derived exosomes for 48 h. RFP+ MDA-231 cells were then added in the transwell insert (8um pores) and cocultured for another 48 h. Top: schematic of experiment. Bottom: quantitation of RFP+ cells among the human hepatocytes. **f** Primary human liver cells were plated for two days, followed by priming with MDA-231-derived exosomes on days 2 and 3. At day 3, RFP+ MDA-231 cells were added to the culture, and 24 h later, the intercalated cells enumerated. Top: schematic of experiment. Bottom: quantitation of RFP+ cells among the liver cells. **g** The experiment in F was evaluated four days after seeding the MDA-231 cells, at the time the primary human liver cells start to die. Top: schematic of experiment. Bottom: quantitation of RFP+ cells. A-D are representative examples of experiments performed at least 3 times, and E-G are mean ± s.e.m. of three experiments each performed in duplicate. * *P* < 0.05

One of our primary goals was to study the effect of the breast oncosomes on the HepN and to assess the potential influence these oncosomes have on the homing, seeding and proliferation of the cancer cells to the metastatic site. Initially we focused on 2D co-culture experiments. After optimization of the exosome isolation from MDA-231 breast cancer cells in 2D cultures we verified the successful exosome harvesting through TEM (Fig. 1a) and immunoblot protein analysis of well-established exosome markers CD63 and CD81 [13, 22] (Fig. 1b). To confirm their functionality, the isolated exosomes were subsequently stained with the red lipophilic dye DiI and introduced to cultures of normal human fibroblasts (TP11/70). After 48 h we observed that the MDA-231 oncosomes were taken up by the fibroblasts and mainly localized in the perinuclear area of the cytoplasm (Fig. 1c), as previously described [23, 24]. Furthermore, in order to monitor the successful incorporation of the MDA-231 exosomes into the hepatocytes in the subsequent 2D co-culture experiments, RFP expressing MDA-231 were transfected with the pCT-CD63-GFP vector and were successfully incorporated into their surrounding hepatocytes (Fig. 1d).

### Priming of the hepatic niche (HepN) with MDA-231 derived exosomes enhances the homing and seeding of the breast cancer cells to the “liver” in 2D co-culture experiments

To evaluate whether the breast cancer cells promote their homing to the metastatic site through exosomes, we established 2D transwell co-culture experiments. Hum Hep/NPCs were plated on the bottom of transwell plates and primed with MDA-231 freshly isolated exosomes for 48 h (20 μg/well, as quantified by BCA assay). Next, 20,000 MDA-231 RFP+ cells were plated on the insert of the transwell plate and were allowed to trans-migrate to the bottom compartment for another 48 h (Fig. 1e, upper panel). After quantification of the RFP+ cells (MDA-231) in the bottom compartment, we observed that oncosomal priming of the HepN resulted in a statistically significant homing of the breast cancer cells to the hepatocyte monolayer (Fig. 1e, lower panel). This indicates that the cancer derived exosomes promote breast cancer cell migration and attachment to the liver by establishing a permissive microenvironment. To decipher whether the oncosomal priming had an effect solely on the migratory potential of the cancer cells, and/or it also confined a survival advantage to the metastasizing cells, enhancing their seeding at the distant site, Hep/NPCs were allowed to attach overnight and were switched to culturing medium containing MDA-231 derived exosomes for 48 h. On day 3, 500 MDA-231 RFP+ breast cancer cells were introduced to the hepatocyte monolayer cultures and on day 4 the experiment was concluded and the percentage of RFP+ area was quantified (Fig 1f). A slightly larger RFP+ area was observed when the HepN was primed with the cancer exosomes; while significance was not attained in this experiment (see below for similar trends), the small increase was reproducible. This suggested that the cancer cells do indeed promote their own homing and attachment/ seeding to the distant site by using exosomes as a means to fertilize the soil for tumor cell dissemination. This is an important finding considering that survival in the new metastatic niche is the most rate-limiting step in the formation of macrometastases.

### Priming of the HepN with MDA-231 derived exosomes promotes cancer cell seeding to the HepN, but results in significantly suppressed cancer cell proliferation in the liver MPS

**Fig. 2:**
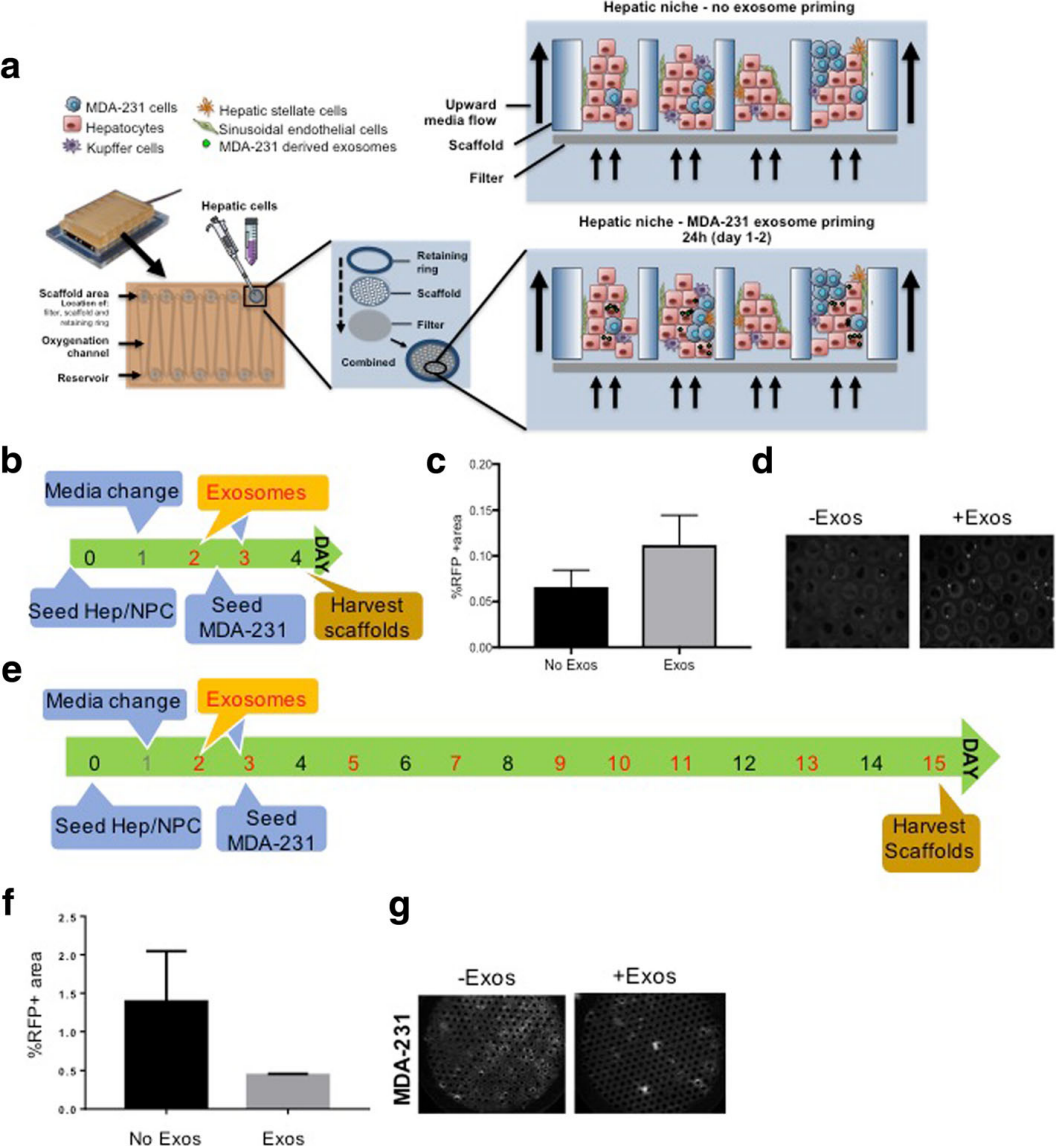
Cancer exosomes enhance seeding but suppress outgrowth in an all human 3D liver tissue. (**a**) Schematic of the liver Microphysiological System (MPS). Magnified view of the wells with (upper right) and without (lower right) priming with MDA-231 derived exosomes. (**b**) Timeline of the experiment to determine seeding efficiency of MDA-231 cells (500 cells) into the liver MPS (total 1.2 million cells). (**c**) Quantification of the RFP+ MDA-231 cells in the MPS 24 h after seeding, with (**d**) representative (right) photomicrographs. (**e**) Timeline of the experiment to determine post-seeding outgrowth in the liver MPS. (**f**) Quantification of the cells 12 days after seeding, with (**g**) representative (right) photomicrographs. C and E are mean ± s.e.m. of three experiments each performed in duplicate. * *P* < 0.05

Our laboratory has introduced the Liver MPS (Fig. 2a), an all-human 3D system (CN Bio Innovations Ltd.; Oxford, UK) that faithfully models both the hepatic niche and micro-metastatic tumor cells [9–11] and we have provided insight into the phenotypic plasticity of both breast and prostate carcinoma cells [2, 3, 8, 25].

We approached this in two ways as with the 2D adherence. First, to assess initial seeding/intercalation, Hum Hep/NPCs were plated on the Liver MPS platform and at days 2 and 3 the wells were primed with 20 μg/well of MDA-231 freshly isolated exosomes (Fig. 2b). On day 3, 500 MDA-231 RFP+ cells were introduced in the system and on day 4 the scaffolds were harvested and the RFP positive area was quantified (Fig. 2c); a representative field is also shown for the RFP fluorescence from the tumor cells (Fig 2d). Thus we are isolating the initial event of intravasation and survival. In the Liver MPS context that closely mimics the in vivo metastatic seeding process, the priming with MDA-231 oncosomes slightly enhanced the tumor cell seeding to the HepN, confirming our observations in the 2D co-culture system. The metastatic site colonization is a necessary step in tumor cell progression and dissemination in the human body and our data indicates that it can be mediated and enhanced by the cancer derived exosomes in a cell autonomous manner.

The second aspect was to determine the fate of the tumor cells in the metastatic environment. Interestingly however, when the breast cancer cells were allowed to proliferate within the Liver MPS for 15 days (Fig. 2e), the initial enhanced seeding we observed above, did not result in an increased breast cancer cell outgrowth. By extending our timeline to 15 days we allowed for the breast cancer cells to outgrow in the metastatic niche through interaction with neighboring cancer cells and surrounding hepatocytes and NPCs. At day 15 the RFP+ area was quantified to extrapolate information on the cancer cell proliferation and was found significantly larger in the wells that had never received oncosomal priming (Fig. 2f, g). This was not only observed in the context of the liver MPS, but the same suppression of tumor cell proliferation was recapitulated in Hep/NPCs 2D co-cultures (Fig. 1g) on day 7 of culture. These results show that the suppression in tumor growth is not due to factors inherent to the Liver MPS. Rather liver-derived signals released in the culture appear to drive this, suggesting a crosstalk between the freshly invading cancer cells that promote their homing and seeding to the liver through exosomes and the receiving hepatic microenvironment.

### Differential exosome protein and miRNA profiles between the HepN, MDA-231 in the HepN and MDA-231 in 2D cultures, identify a novel contribution of the HepN to the cancer cells during metastatic outgrowth

The discrepancy between tumor cell seeding and tumor cell proliferation after oncosomal priming, prompted us to search for a potential cause. At this point it is unclear however, whether this decrease in the proliferation levels and tumor cell outgrowth could be attributed to the effect of cancer derived exosomes, on the metastasizing cancer cells through selection for less proliferative, more quiescent clones, and/or due to the interaction of the activated Hep/NPCs with the cancer cells with which they are now in close proximity. And despite the fact that the tumor derived exosomes have been extensively studied, little is known about the normal tissue (e.g. HepN) derived exosomes.

**Fig. 3:**
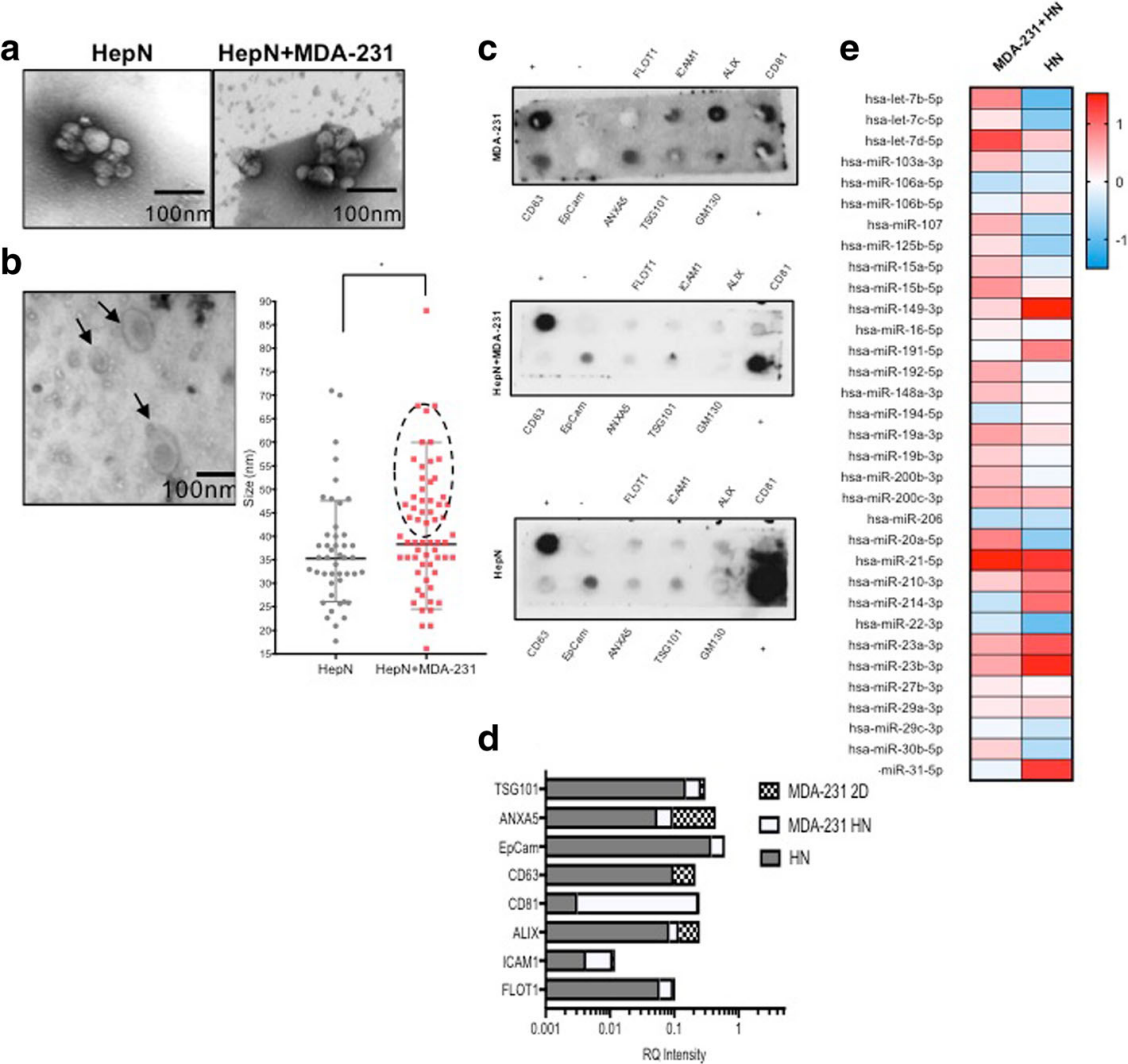
Exosomes are produced by the liver MPS. **a** Transmission electron microscopy (TEM) of concentrated effluent from the liver MPS with or without 500 MDA-231 cells within the 1.2 million liver cells. HepN = Hepatic Niche (includes hepatocytes and non-parenchymal cells). **b** Exosomes from the MPS with breast cancer cells have extracellular vesicles of varied sizes. Left: representative TEM, Right: quantitation of exosome size (maximum diameter in nm). **c** Exosome protein arrays performed on exosome protein extracts from HepN and HepN + MDA-231 in the Liver MPS and MDA-231 in 2D cultures, with (**d**) relative quantification. **e** Analysis of miRNA content of the exosomes isolated from the liver MPS. Shown are representative examples of experiments performed at least 3 times

After isolating exosomes from the liver MPS, we observed that the hepatic niche alone contributes significantly to the total exosome pool (Fig. 3a). However, this was expected since the number of MDA-231 that are introduced into the hepatic microenvironment in the Liver MPS is limited (only 500 cells per well) and even though cancer cells are known to produce significantly larger numbers of exosomes [13, 26] a ratio of 1:4 × 10^4^ would still be hard to overcome. Furthermore, we observed that in the samples derived from the Liver MPS there was a variability in the exosome size (Fig. 3b). The average size (as quantified by exosome diameter) was statistically significantly different in the 2 groups (HepN: 35.4 nm, MDA-231 + HepN: 38.7 nm) and we can easily identify a subgroup of exosomes in the MDA-231 + HepN group with a size of 40-75 nm which we hypothesize is originating from the MDA-231 cells, as it has previously been proposed that oncosomes’ size is larger than the normal tissue derived exosomes [27, 28].

Exosome protein analysis was performed using an exosome specific antibody array, and showed that the protein expression pattern in the three groups (MDA-231 alone in 2D, MDA-231 + HepN in the Liver MPS and HepN alone in the Liver MPS) differed significantly (Fig. 3c). Even though for most of the proteins tested the expression levels in the exosomes of MDA-231 + HepN were distinct from the MDA-231 alone, these differences cannot be attributed to a mere additive contribution of the HepN derived exosomes in the pool of the total exosomes isolated from the MDA-231 + HepN sample. Along these lines, after normalization of all the samples to internal controls, proteins like FLOT1, EpCam, TSG101 and ANXA5 had a very similar expression pattern in the HepN and MDA-231 + HepN samples pointing to the direct contribution of the HepN to exosomes isolated. But other proteins like CD81, CD63 and ALIX showed a dramatic difference between the two groups and also differed significantly from the MDA-231 derived exosomes (Fig. 3d), hinting towards a HepN mediated cancer cell transformation. In accordance to these findings, comparing the exosome miRNA content of the HepN alone versus the HepN + MDA-231, the miRNA content of the latter did not faithfully follow the expression pattern of the HepN alone (Fig. 3e). [Relative levels of the miRNA are provided in Additional file 1: Figure S1.] Our data collectively showed that within the micrometastatic milieu the two exosome subgroups (normal and cancer) co-exist and co-operate to modify the genotypic and phenotypic characteristics of the metastasizing cancer cells during the metastatic tumor seeding and outgrowth phase.

### The hepatic microenvironment suppresses tumor cell proliferation by exosome mediated signal transfer

**Fig. 4:**
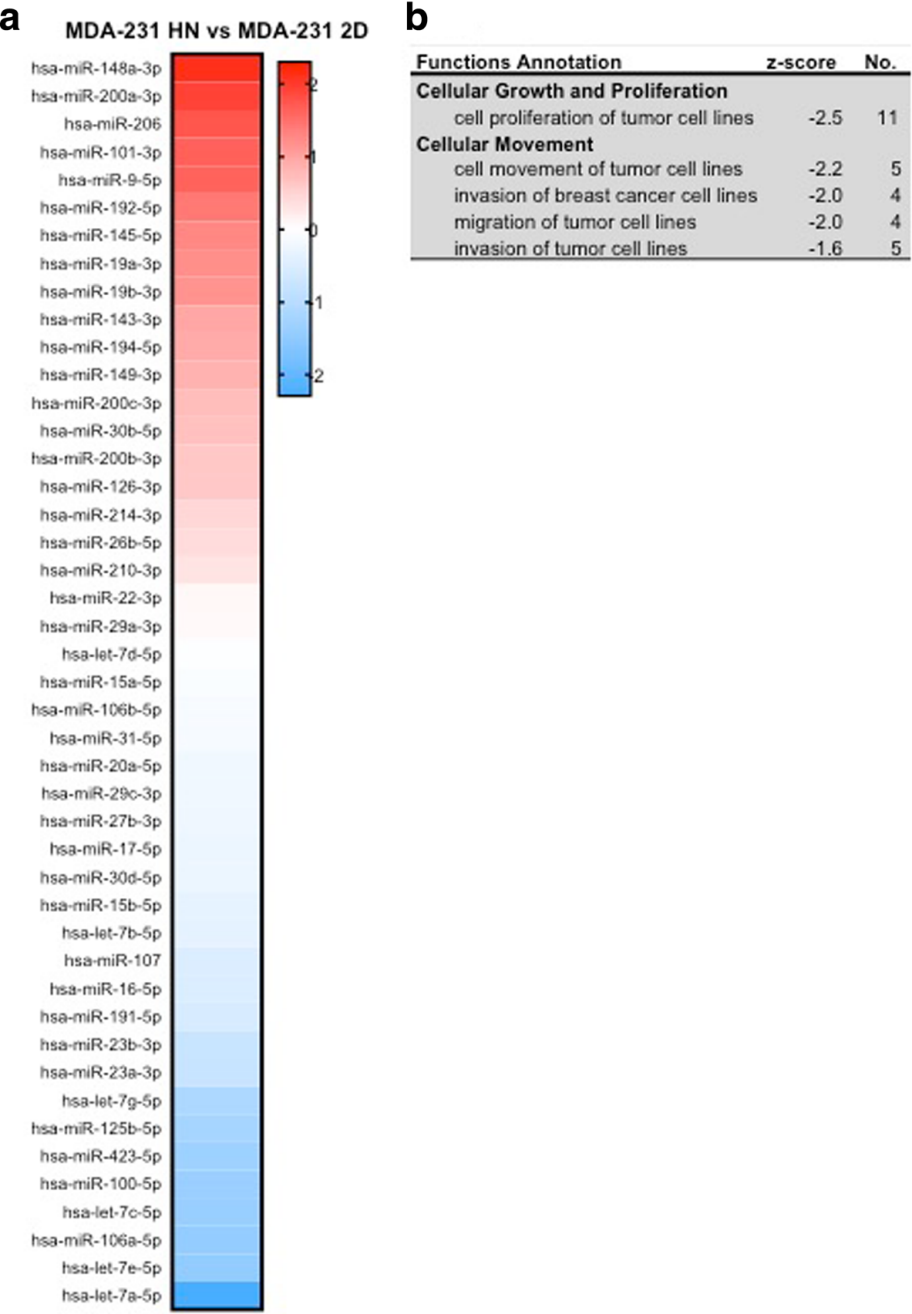
Comparison of miRNA in exosomes derived from HepN with MDA-231 cells versus those from 2D cultures of MDA-231 cells (**a**). **b** The Ingenuity Pathway Analysis (IPA) suggests a targeting of pathways involved in cell growth and locomotion. Results are from three independent experiments

In order to further elucidate the effect of the cancer cell and hepatic exosomes intercommunication, we isolated exosomes from MDA-231 in 2D cultures and MDA-231 from the liver MPS and a comparative analysis of their miRNA content was performed (Fig. 4a). [Relative levels of the miRNA are provided in Additional file 1: Figure S2]. The differential miRNA profiles were then analyzed using the IPA software and the activation z-score of transcriptional regulators showed that MDA-231 in the HepN had significantly suppressed the pathways promoting cancer cell proliferation and invasion (z-score < −2) (Fig. 4b). This finding correlates with the limited proliferation rates that we observed in the Liver MPS above, but one could argue that it is an epiphenomenon due to the large number of HepN derived exosomes in the total pool of exosomes isolated from the liver MPS.

To determine the effect of the HepN on cancer cell proliferation, after the initial cancer cell seeding, 2D co-culture experiments of HepN with melanoma cells (WM35, WM852 and TPF16238) were prepared. After the initial 48 h priming with the respective melanoma derived exosomes, the cancer cells were introduced to the hepatocyte monolayer on day 3 and were allowed to proliferate until day 7. On day 7 the cultures and supernatants were collected for cell cycle analysis using PI which revealed that in all three lines tested there was an increase in the cells that were in G0-G1 phase with a minimal change in the actively dividing cell populations (G2/M and S phase) (Additional file 1: Figure S3A). The decrease in the cells in SubG0 phase of the cell cycle indicates that after priming of the HepN with the cancer derived exosomes, the HepN signals to the cancer cells to help them avoid cell death during colonization to the liver and promotes a slow entry into the cell cycle and a proliferative arrest. This also shows that the effect of the HepN is not cell type specific and is not limited to the breast cancer cells. Exosomes purified from the HepN in the Liver MPS were analyzed for their miRNA content and even though no pathways were overall found suppressed or activated, there was a significant enrichment in miRNAs involved in cell cycle regulation and more specifically cancer cell arrest in G1 phase (Additional file 1: Figure S3B).

### Purified HepN derived exosomes promote changes in the cancer cells consistent with a partial MErT

**Fig. 5:**
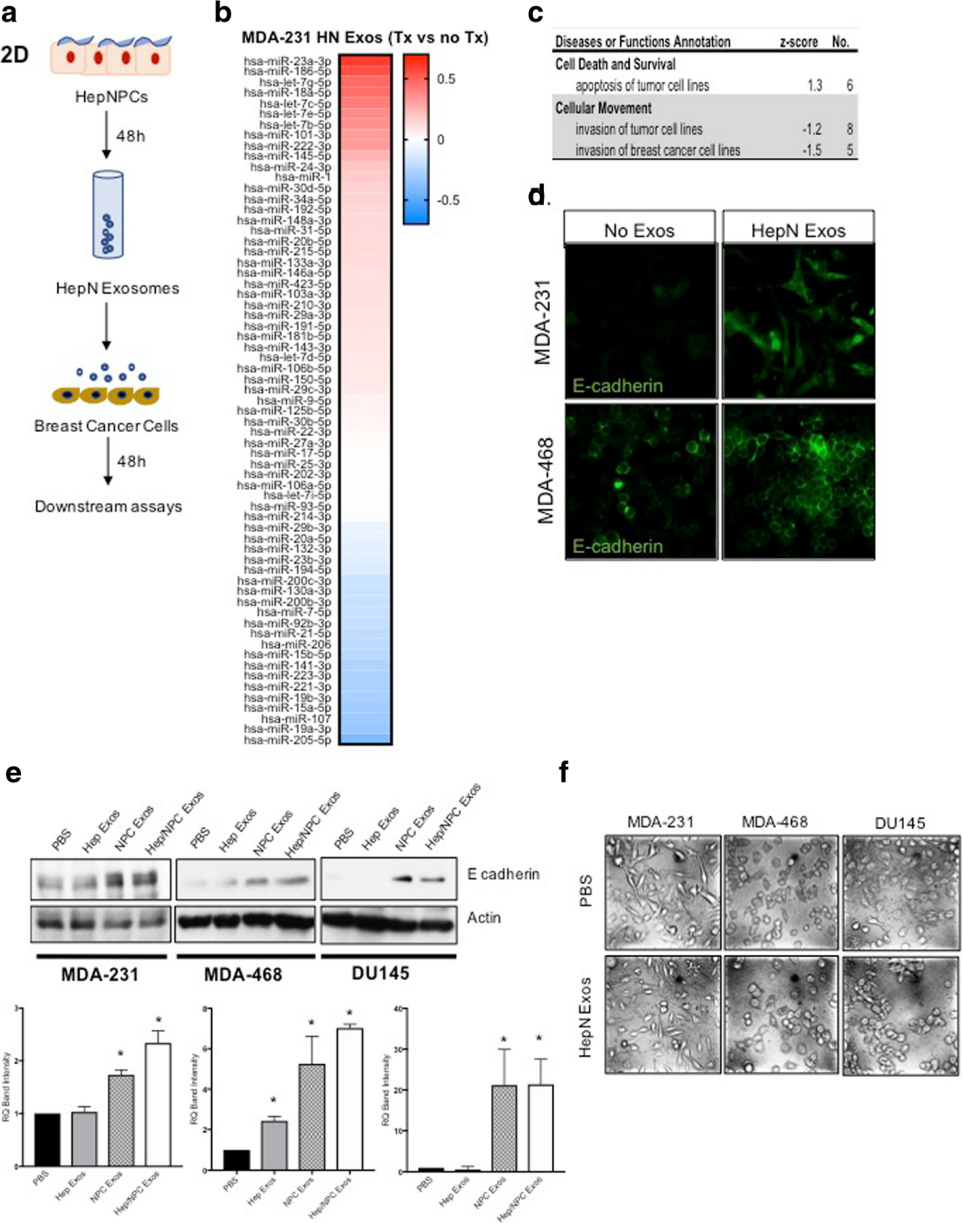
Hepatic Niche-derived exosomes quiesce cancer cells. (**a**) Schematic of the experimental outline in which HepN exosomes are collected and used to prime cancer cells. (**b**) Analysis of the miRNA content of MDA-231 that received treatment with HepN derived exosomes for 48 h versus MDA-231 treated with PBS alone shows a difference in those controlling pathways survival and locomotion of cancer cells (**c**) as suggested by IPA. (**d**) Immunofluorescence shows an upregulation of the epithelial marker E-cadherin in MDA-231 cells and membrane cohesion of E-cadherin in the MDA-468 cells. (**e**) This is corroborated by immunoblotting (upper are representative blots, lower is quantitation), demonstrating that the exosomes from the NPC provide most of the impetus. [Note: Immunoblot for MDA-231 E-cadherin is overexposed to detect lower levels of E-cadherin compared to the other lines.] (**f**) Phase microscopy demonstrates more epithelial morphology at least in the DU145 and MDA-468 cells. Representative experiments of at least three are shown

To dissociate the specific role of the HepN derived exosomes on the cancer cells versus the paracrine and autocrine effects of soluble factors secreted in the culture medium, we isolated pure exosomes from 2D cultures of Hep/NPCs and used them to treat MDA-231 cells for 48 h (Fig. 5a). Upon analysis of the miRNA content of MDA-231 treated with hum HepN exosomes vs controls treated with vehicle PBS, we saw that the pathways that were significantly downregulated were implicated in cancer cell invasion (z-score < −1.2) (Fig. 5b, c). [Relative levels of the miRNA are provided in Additional file 1: Figure S4] In the panel of miRNAs that we tested several of the miRNAs that were significantly enriched or suppressed in the MDA-231 cells treated with HepN exosomes have previously been reported to suppress cancer cell invasion and modulate E-cadherin expression. In numerous cancers miR-186 has been shown to inhibit cell proliferation and act as an antioncogenic miR [28–30] furthermore previous work from Zhu et al. shows that miR-186 can promote MErT by enhancing E-cadherin expression and suppressing vimentin [31] Upregulation of the let-7 family has been shown to be involved cancer cell differentiation, and to inhibit liver metastasis by >50%. From the top downregulated miRNAs, miR205 has been reported as a negative regulator of E-cadherin levels, promoting, through Snail upregulation, a more proliferative and invasive phenotype [32] and silencing of miR-19 has been described to reverse EMT in lung cancer by suppressing E-cadherin [33].

Our laboratory in previously published work has shown that MDA-231, which are negative for E-cadherin could be induced to re-express it by in vitro coculture with normal hepatocytes or following spontaneous metastasis to the lung in a mouse model [3, 8]. To investigate whether this effect could potentially be mediated by transfer of HepN derived exosomes to the cancer cells we treated MDA-231 and MDA-468 cancer cells with pure HepN derived exosomes. Using immunofluorescence we observed that the wells that had received HepN exosomes significantly upregulated the levels of E-cadherin. We were able to reproduce the induction of E-cadherin in both breast cancer lines and the prostate cancer line DU145 (Fig. 5e). The induction of E-cadherin was not accompanied by a significant decrease in vimentin levels or levels of mRNA for Twist, snail or slug (data not shown) but ZO-1 was upregulated in two of the three lines (Additional file 1: Figure S5). Changes in E-cadherin levels during MErT are driving changes in cell morphology from a mesenchymal to a more epithelial type, which was indeed the case for MDA-468 and DU145 (Fig. 5f). No changes were observed in the morphology of MDA-231 cells after HepN exosome treatment potentially due to increased autocrine downregulation.

## Discussion

Metastasis is a multistep complex process that despite having detrimental effects on the patient’s prognosis is inherently highly inefficient. In order to metastasize, the cancer cells need to undergo EMT to escape the primary tumor, extravasate into the circulation, avoid cell death due to lack of support from the surrounding cancer mass and extracellular matrix and subsequently colonize the distant site and resume proliferation. Many of these steps have been well studied and elucidated but recent advances have added a new level of complexity by identifying the cancer derived exosomes as critical shuttles for cell to cell communication, promoting tumor progression and increasing the efficacy of the metastatic cascade [13]. Tumor derived exosomes mediate the transfer of oncogenic proteins and nucleic acids thus inducing a persistent and efficient modulation in the genotype and phenotype of the recipient cells [34–36]. Furthermore, they promote the recruitment and reprogramming of the tumor microenvironment to form a pro-tumorigenic soil, by establishing a positive feedback loop that shapes the ever-evolving tumor microenvironment and allows cancer cell seeding, proliferation and dissemination at distant sites [37–39]. However, the specific mechanisms through which healthy cells are triggered to release exosomes that promote or suppress the malignant behavior of cancer cells remain to be determined.

It must be noted that while we find classical exosomes, and our preparations appear mainly to consists of these as noted by classical shape and size on electron microscopy, the isolation techniques will enrich for all extracellular vesicles, both the larger blebs and the smaller exomeres. Herein we are not distinguishing between these, but rather using exosomes as the shorthand to encompass all such communicating vesicles.

Using our Liver MPS to closely reproduce the events of the metastatic cascade (ref BJC and LoC papers), we show that priming of the hepatic microenvironment with cancer derived exosomes promotes the seeding of breast cancer cells to the metastatic site. This is in agreement with published literature that shows the effect of the oncosomes in establishing a pre-metastatic niche [37–39]. However, more importantly we were able to recognize the significant contribution of the normal HepN derived exosomes to the exosomal pool during metastasis. It should be noted that the source of liver cells comes from donors undergoing partial hepatectomies for both benign and malignant diseases (though the cells are obtained from tissue distant from the pathology). While we do not note differences in MPS behavior between these sources, it is possible the pathology necessitating the partial hepatectomy influences the exosome profile and cargo even weeks later in the MPS. The HepN derived exosomes differ from the cancer derived exosomes in their size and their miRNA and protein cargo. These HepN derived exosomes are produced constantly but become more relevant once the cancer cells reach the liver microenvironment as they are now within range of interaction. It is also important to keep in mind that the cancer cells could potentially affect the normal cells through exosomes and soluble factors, modifying the exosome pool that the latter secrete. Herein we show that after the initial seeding to the liver, the cancer cells communicate with the HepN through exosomes and via miRNA transfer, the hepatic microenvironment suppresses cancer cell proliferation and promotes slow cell cycling.

These studies are performed ex vivo which entails both advantages and limitations. The all-human situation cannot be recapitulated in animal models, and thus select signals or miRNA that do not cross species may be approachable only with such a MPS. Further, the isolation of compartments and tissues, with rapid sampling of unmodified effluent is not readily achieved in vivo. Still, once specific molecules are identified as causally required, these may be targetable in animal models of spontaneous metastasis in future investigations.

Dormancy, defined as a more quiescent cell state, marked by slow proliferation and low metabolic activity has proven to be the main cause of treatment failures and cancer relapses. IPA of the miRNA profile of the MDA-231 in HepN shows a significant suppression in the pathways involved in cancer cell proliferation and invasion indicating that the interaction with the liver microenvironment results in alterations in the MDA-231 expression profile and changes in the cancer cell phenotype. To corroborate these findings we performed cell cycle analysis of 2D co-cultures and showed a delay of the cells in the G0-G1 phase and that the HepN derived exosomes are enriched in miRNAs involved in the regulation of G1 arrest. In the exosome protein array we observe a significant elevation of the exosomal marker CD81 in the MDA-231 cells in the HepN. The tetraspanin CD81 has been reported to be important regulator of cell fate in hematopoietic stem cells (HSC) and CD81 can drive proliferating HSC to quiescence [40]. The HepN derived exosome-induced suppression of cancer cell proliferation is potentially a “tumor suppressive” mechanism that the normal tissue microenvironment at the distant site utilizes in order to maintain a steady state and minimize the changes that occur within it after the arrival and seeding of the cancer cells, however in the long term this could have detrimental effects for the patient since the cells can resume proliferation and outgrow at any time in the future. This observation has significant clinical implications considering that the cell cycle status often determines the cell’s response to chemotherapeutic agents. Future studies are required to further characterize the genotypic and phenotypic changes that occur and to further validate the dormant state of the cancer cells and their potentially acquired chemoresistance.

Furthermore we have previously shown that interaction of the breast cancer cells with the hepatocytes and NPCs in the liver leads to a re-expression of E-cadherin and a partial MErT [3, 8]. Our initial studies had found that neither conditioned media alone nor hepatocyte-derived matrix could trigger E-cadherin re-expression in this breast carcinoma line, though the combination of the two was noted to lead to a weak re-expression of E-cadherin [8]. Herein we provide evidence that this effect on the breast cancer cells is exosome mediated and that treatment of breast and prostate cancer cells with purified normal HepN derived exosomes increases E-cadherin and ZO-1 protein expression levels and induces a more epithelial-like morphology. No significant changes were observed in the levels of the mesenchymal marker Vimentin suggesting a partial MErT. Our findings highlight the importance of the exosomes derived from the receptive microenvironment in the phenotypic plasticity of the cancer cells during tumor progression and the metastatic cascade. Through MErT and expression of adhesion molecules, the reversible phenotypic plasticity allows cancer cells to adapt to the foreign soil during ectopic organ colonization [41]. Expression of the epithelial-marker and the cell adhesion molecule E-cadherin on breast cancer cells may be another mechanism to facilitate adhesion to hepatocytes [24].

The effects that we observe could largely be attributed to the exosomal transfer of miRNAs as these are master regulators. By comparing the miRNA content of MDA-231 that were exposed to HepN derived exosomes to MDA-231 we see that the miRNAs that are significantly upregulated or downregulated have been reported previously to regulate E-cadherin expression and MErT in various cancer types [28–36]. Additional testing of our miRNA hits in the top and bottom bins is essential for the identification of E-cadherin and MErT transcriptional regulators in our system since targeting with antagomirs could be a useful clinically applicable method to target the downstream pathways. Furthermore it is known that in most carcinomas, and namely in the MDA-231 breast cancer line, E-cadherin appears to be turned off at the translational level by promoter hypermethylation [42, 43]. Aberrations of DNA methylation have been inferred to play an important role during the initiation and progression of various cancers [44] and they could potentially play a critical role in our system as well since exosomes can act as epigenetic reprogrammers [45]. Definitive assignment of critical role of likely multiple and overlapping miRNA will require both defining the cell types of origin and then targeted disruption, both of which lie beyond the scope of the present missive.

## Conclusions

Our data collectively identifies a novel mechanism of regulation of the metastatic cascade, introducing the normal tissue/HepN derived exosomes as significant modulators of MErT during seeding and suppression of tumor growth once the breast cancer cells have reached the liver. The importance of the normal exosomes during the metastatic cascade has been widely underestimated, however we show that they become critical players during metastasis. We highlight the presence of a well-orchestrated exosome mediated bidirectional crosstalk between the cancer cells and the HepN that differentially regulates the steps of the metastatic cascade. The importance of the exosomes as signal shuttles stems from their newly established close proximity with the cancer cells. The concentration of exosomes in the blood are likely too dilute to strongly signal the liver from the primary site, and particularly in reverse. However, once the disseminated cell intravasates into the metastatic locale, the microenvironmental concentrations of these signals would be magnified, and likely more effective in altering cellular and tissue phenotypes. The present study posited such a directed bidirectional interaction. For the first time we introduce evidence that not only shows that cancer derived exosomes modify the premetastatic niche but the metastatic niche in turn also modulates the subsequent steps of the metastatic cascade determining the cancer cell fate.

## Additional file

Additional file 1: Figure S1. Relative absolute values of the miRNA species are provided for comparison to each other coming from the bioreactor with and without MDA-231 cells. **Figure S2.** Relative absolute values of the miRNA species are provided for comparison to each other coming from the MDA-231 cells in 3D tissue versus 2D. **Figure S3.** A upper panel: Experimental outline for cell cycle analysis. A lower panel: Cell cycle analysis, using Propidium Iodide, of 2D co-culture experiments, following priming of the hepatic niche with cancer derived exosomes. B: miRNA content of exosomes derived from the HepN in the Liver MPS. C: IPA analysis of the miRNA content in the exosomes described above. **Figure S4.** Relative absolute values of the miRNA species are provided for comparison to each other coming fromMDA-231 cells after treatment with HN exosomes. **Figure S5.** Western blot analysis for ZO-1 protein levels on the breast cancer lines MDA-231 and MDA‐468 and the prostate cancer line DU-145 that were treated exosomes derived from human hepatocytes, NPCs and Hep/NPCs for 48 h. Cell prolifera8on of MDA-231 cells treated with exosomes derived from human hepatocytes, and Hep/NPCs for 48 h, quan8fied by trypan blue. Transwell migration of MDA-231 cells treated with exosomes derived from human hepatocytes, and Hep/NPCs for 48 h. (PPTX 540 kb)
