# Supplementary material for: Bi-directional exosome-driven intercommunication between the hepatic niche and cancer cells
Source: Mol Cancer. 2017 Nov 14;16:172. doi: 10.1186/s12943-017-0740-6 (PMC5686836; doi:10.1186/s12943-017-0740-6)
Supplement: Additional file 1: Figure S1. — Relative absolute values of the miRNA species are provided for comparison to each other coming from the bioreactor with and without MDA-231 cells. Figure S2. Relative absolute values of the miRNA species are provided for comparison to each other coming from the MDA-231 cells in 3D tissue versus 2D. Figure S3. A upper panel: Experimental outline for cell cycle analysis. A lower panel: Cell cycle analysis, using Propidium Iodide, of 2D co-culture experiments, following priming of the hepatic niche with cancer derived exosomes. B: miRNA content of exosomes derived from the HepN in the Liver MPS. C: IPA analysis of the miRNA content in the exosomes described above. Figure S4. Relative absolute values of the miRNA species are provided for comparison to each other coming fromMDA-231 cells after treatment with HN exosomes. Figure S5. Western blot analysis for ZO-1 protein levels on the breast cancer lines MDA-231 and MDA‐468 and the prostate cancer line DU-145 that were treated exosomes derived from human hepatocytes, NPCs and Hep/NPCs for 48 h. Cell prolifera8on of MDA-231 cells treated with exosomes derived from human hepatocytes, and Hep/NPCs for 48 h, quan8fied by trypan blue. Transwell migration of MDA-231 cells treated with exosomes derived from human hepatocytes, and Hep/NPCs for 48 h. (PPTX 540 kb) [file 12943_2017_740_MOESM1_ESM.pptx]

## Slide 1
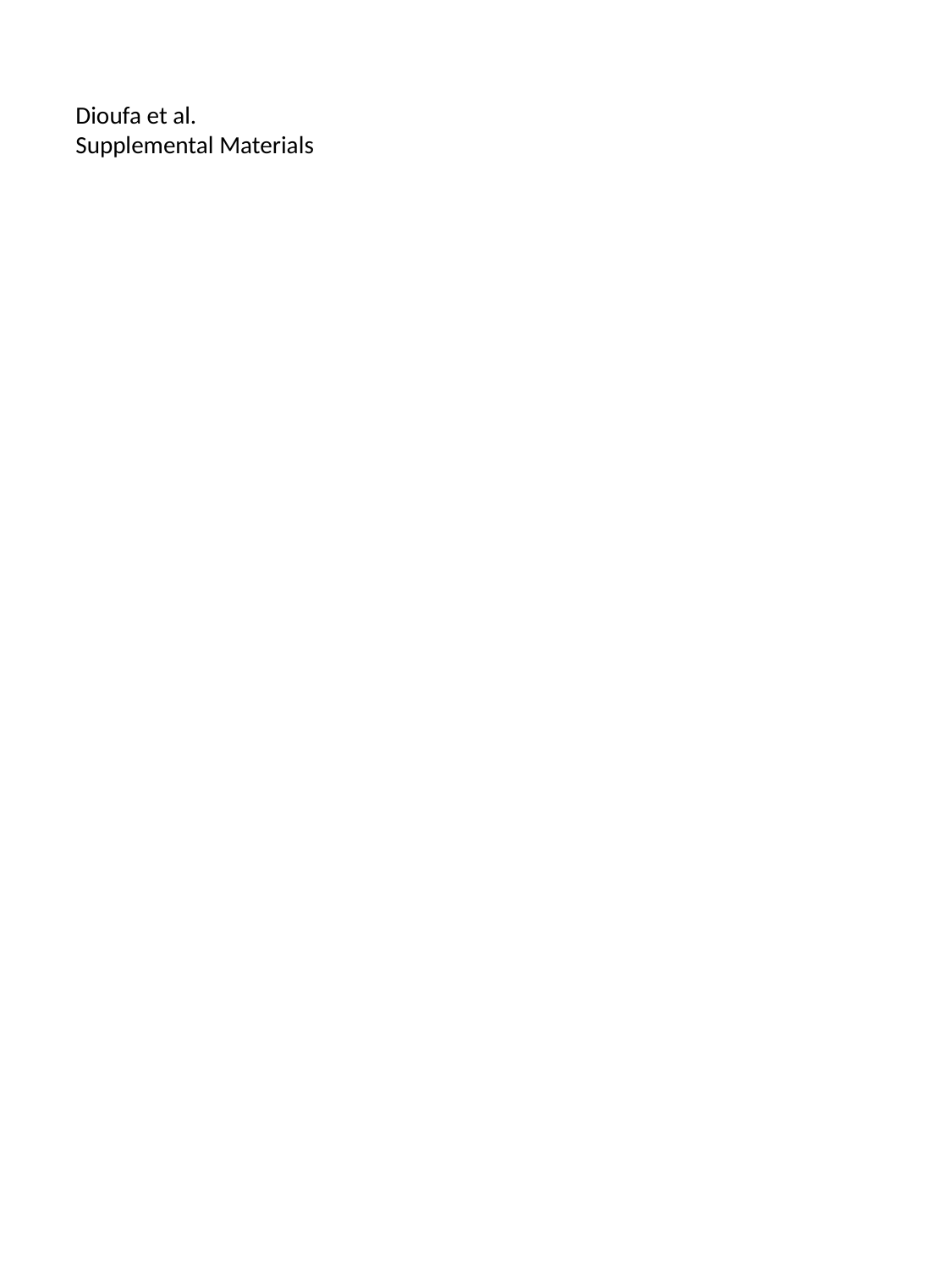

Dioufa et al.
Supplemental Materials

## Slide 2
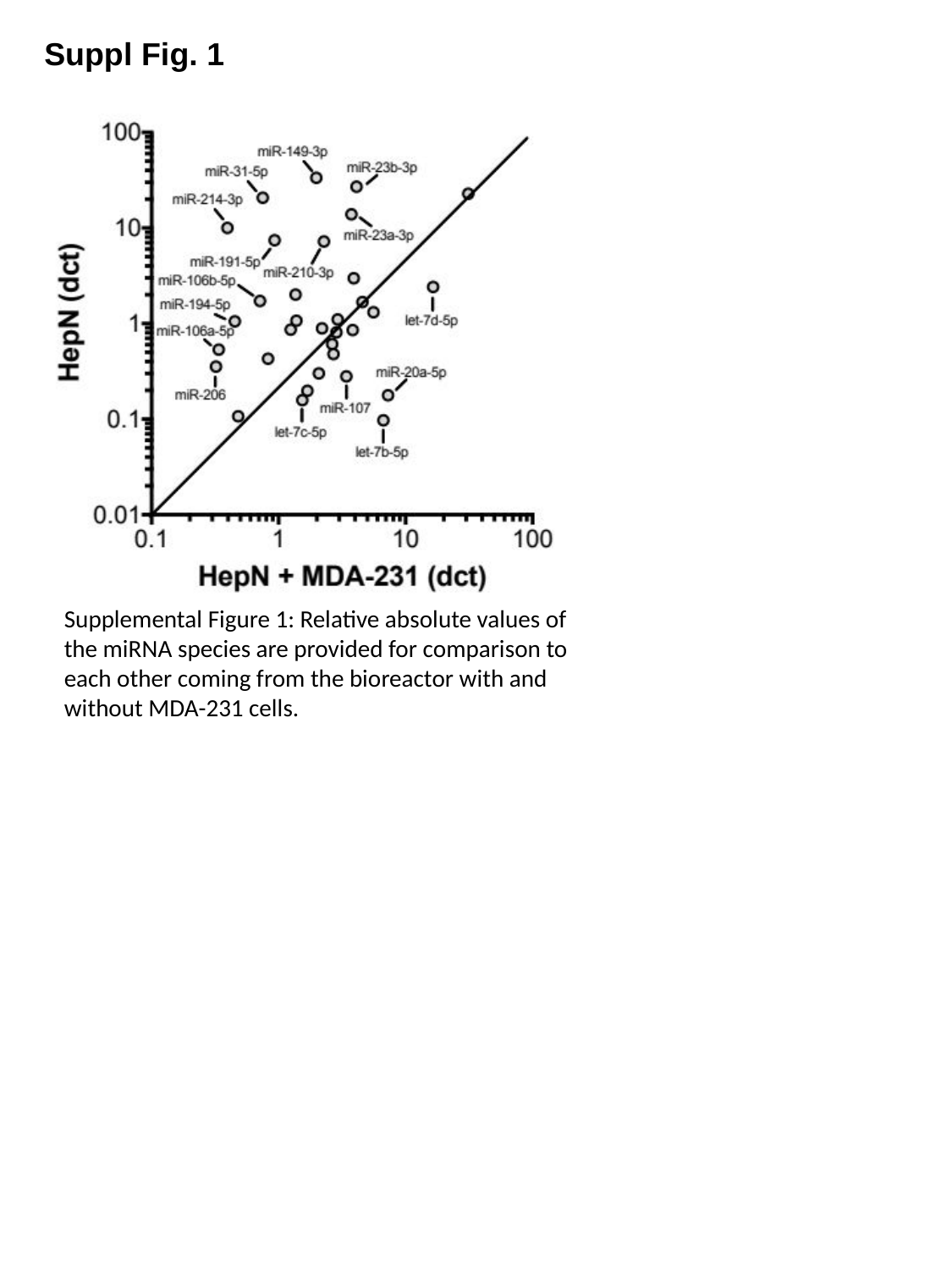

Suppl Fig. 1
Supplemental Figure 1: Relative absolute values of the miRNA species are provided for comparison to each other coming from the bioreactor with and without MDA-231 cells.

## Slide 3
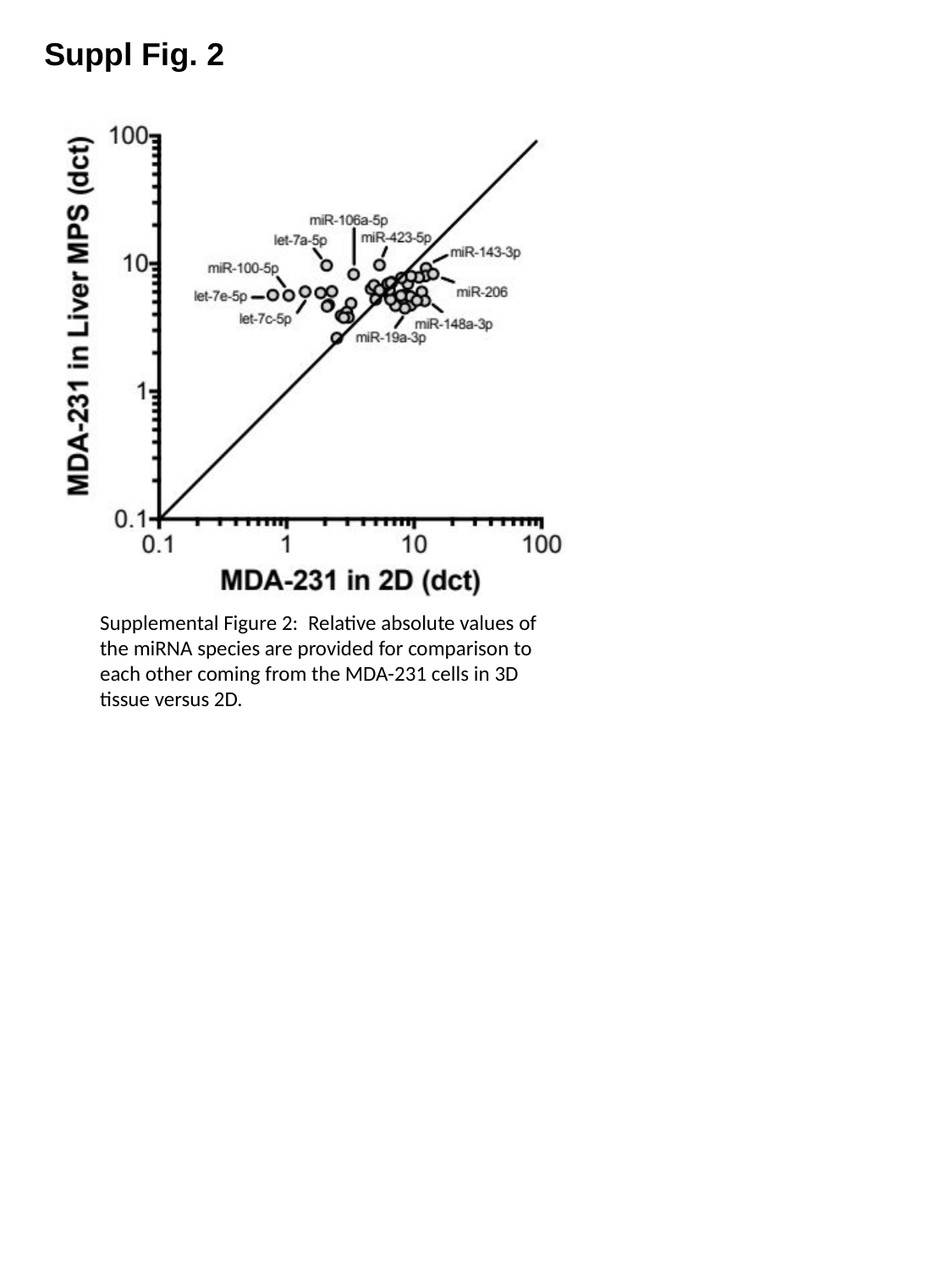

Suppl Fig. 2
Supplemental Figure 2: Relative absolute values of the miRNA species are provided for comparison to each other coming from the MDA-231 cells in 3D tissue versus 2D.

## Slide 4
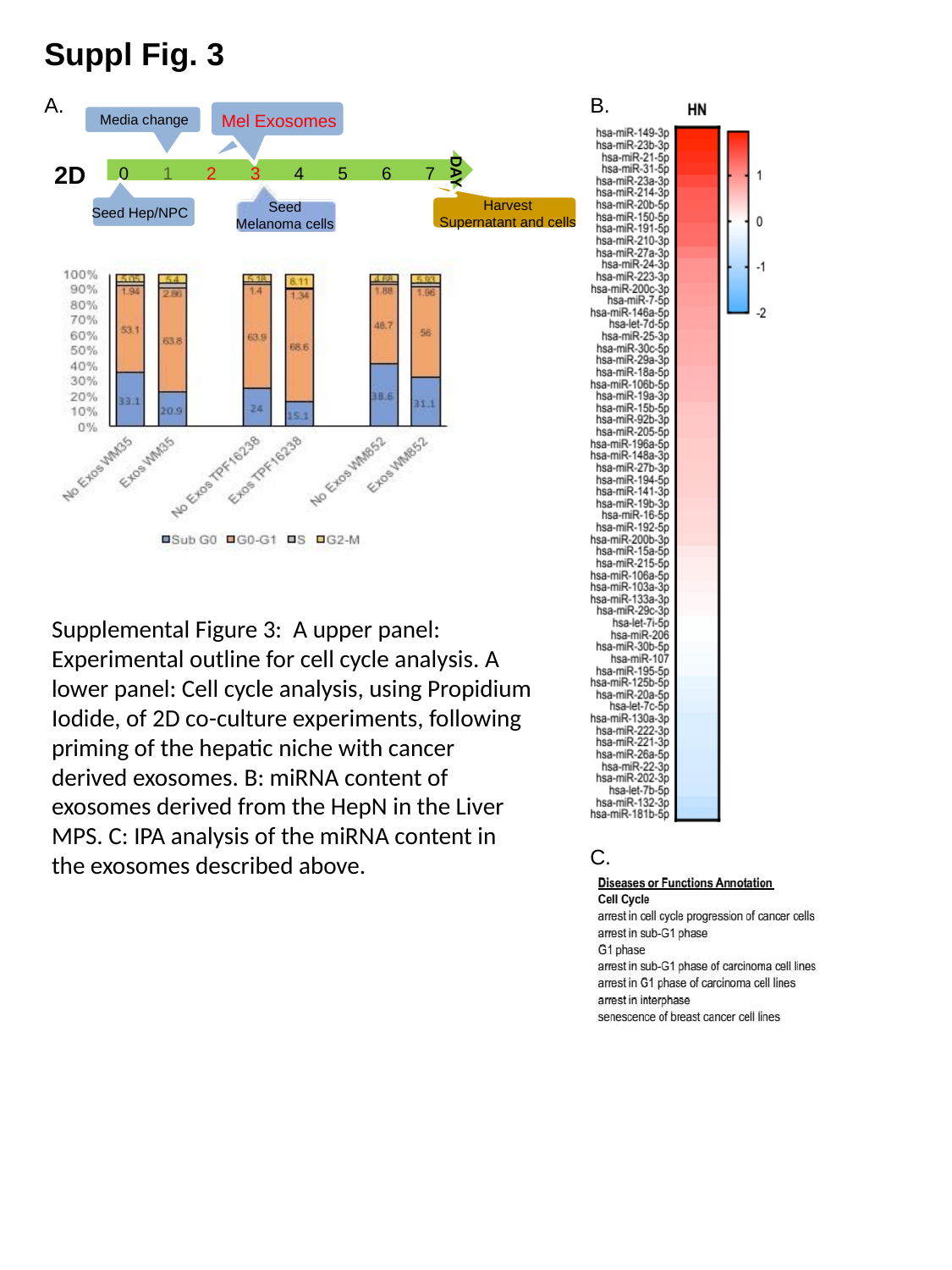

Suppl Fig. 3
B.
A.
Mel Exosomes
Media change
0 1 2 3 4 5 6 7
DAY
Harvest
Supernatant and cells
Seed
Melanoma cells
Seed Hep/NPC
2D
Supplemental Figure 3: A upper panel: Experimental outline for cell cycle analysis. A lower panel: Cell cycle analysis, using Propidium Iodide, of 2D co-culture experiments, following priming of the hepatic niche with cancer derived exosomes. B: miRNA content of exosomes derived from the HepN in the Liver MPS. C: IPA analysis of the miRNA content in the exosomes described above.
C.

## Slide 5
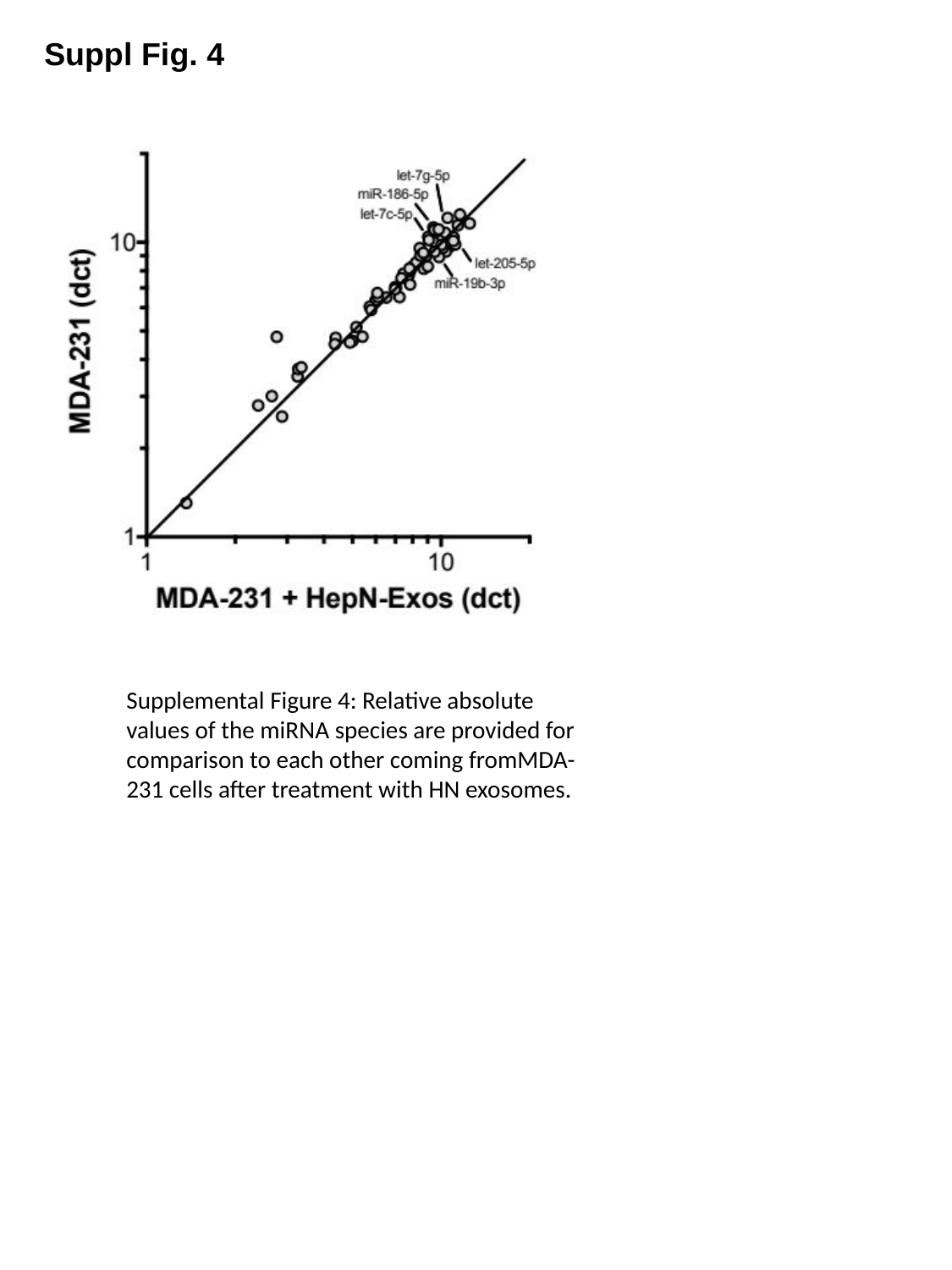

Suppl Fig. 4
Supplemental Figure 4: Relative absolute values of the miRNA species are provided for comparison to each other coming fromMDA-231 cells after treatment with HN exosomes.

## Slide 6
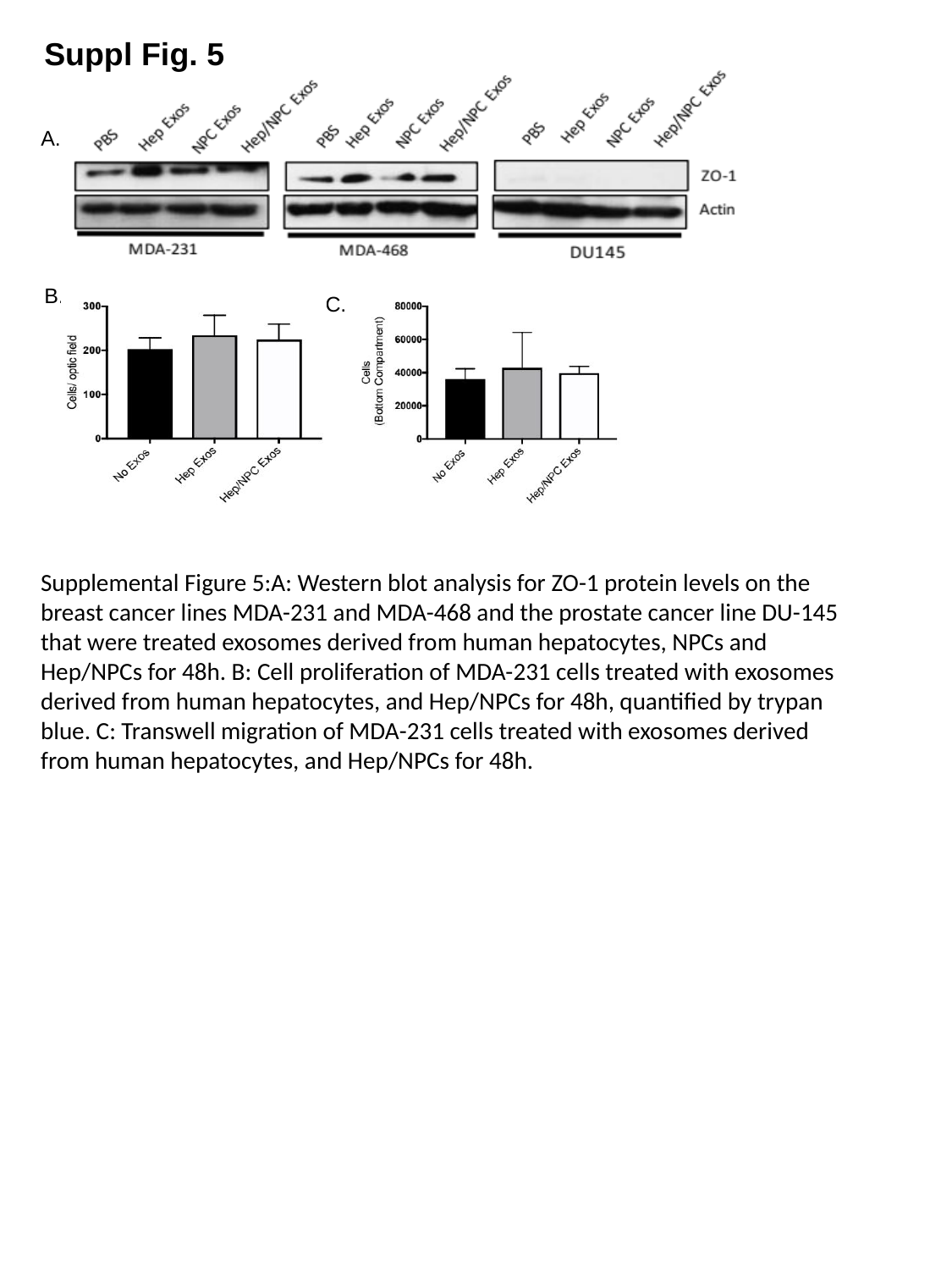

Suppl Fig. 5
A.
B.
C.
Supplemental Figure 5:A: Western blot analysis for ZO-1 protein levels on the breast cancer lines MDA-231 and MDA-468 and the prostate cancer line DU-145 that were treated exosomes derived from human hepatocytes, NPCs and Hep/NPCs for 48h. B: Cell proliferation of MDA-231 cells treated with exosomes derived from human hepatocytes, and Hep/NPCs for 48h, quantified by trypan blue. C: Transwell migration of MDA-231 cells treated with exosomes derived from human hepatocytes, and Hep/NPCs for 48h.
